# Ultrasound-directed enzyme-prodrug therapy (UDEPT) using self-immolative doxorubicin derivatives

**DOI:** 10.7150/thno.69168

**Published:** 2022-06-06

**Authors:** Karolin Roemhild, Helena C. Besse, Bi Wang, Quim Peña, Qingxue Sun, Daiki Omata, Burcin Ozbakir, Clemens Bos, Hans W. Scheeren, Gert Storm, Josbert M. Metselaar, Haijun Yu, Ruth Knüchel-Clarke, Fabian Kiessling, Chrit T.W. Moonen, Roel Deckers, Yang Shi, Twan Lammers

**Affiliations:** 1 Department of Nanomedicine and Theranostics, Institute for Experimental Molecular Imaging, Uniklinik RWTH Aachen and Helmholtz Institute for Biomedical Engineering, Faculty of Medicine, RWTH Aachen University, 52074 Aachen, Germany.; 2 Institute of Pathology, Uniklinik RWTH Aachen, Faculty of Medicine, RWTH Aachen University, 52074 Aachen, Germany.; 3 Division of Imaging and Oncology, University Medical Center Utrecht, 3584 CX Utrecht, the Netherlands.; 4 Department of Pharmaceutics, Utrecht University, Utrecht, 3584 CG, The Netherlands.; 5 Laboratory of Drug and Gene Delivery Research, Faculty of Pharma-Science, Teikyo University, Tokyo, Japan.; 6 Department of Targeted Therapeutics, University of Twente, Enschede, The Netherlands.; 7 Department of Surgery, Yong Loo Lin School of Medicine, National University of Singapore, 119074 Singapore, Singapore.; 8 State Key Laboratory of Drug Research & Center of Pharmaceutics, Shanghai Institute of Materia Medica, Chinese Academy of Sciences, Shanghai, 201203, China.

**Keywords:** Cancer, Prodrugs, Focused ultrasound, HIFU, β-glucuronidase

## Abstract

**Background:** Enzyme-activatable prodrugs are extensively employed in oncology and beyond. Because enzyme concentrations and their (sub)cellular compartmentalization are highly heterogeneous in different tumor types and patients, we propose ultrasound-directed enzyme-prodrug therapy (UDEPT) as a means to increase enzyme access and availability for prodrug activation locally.

**Methods:** We synthesized β-glucuronidase-sensitive self-immolative doxorubicin prodrugs with different spacer lengths between the active drug moiety and the capping group. We evaluated drug conversion, uptake and cytotoxicity in the presence and absence of the activating enzyme β-glucuronidase. To trigger the cell release of β-glucuronidase, we used high-intensity focused ultrasound to aid in the conversion of the prodrugs into their active counterparts.

**Results:** More efficient enzymatic activation was observed for self-immolative prodrugs with more than one aromatic unit in the spacer. In the absence of β-glucuronidase, the prodrugs showed significantly reduced cellular uptake and cytotoxicity compared to the parent drug. High-intensity focused ultrasound-induced mechanical destruction of cancer cells resulted in release of intact β-glucuronidase, which activated the prodrugs, restored their cytotoxicity and induced immunogenic cell death.

**Conclusion:** These findings shed new light on prodrug design and activation, and they contribute to novel UDEPT-based mechanochemical combination therapies for the treatment of cancer.

## Introduction

Prodrugs are widely used in healthcare. They are designed to alter the pharmacokinetics, bioavailability and/or toxicity of drug molecules 1. Chemical, physical and enzymatic stimuli can activate prodrugs, eventually converting them to the active parent drug 2. A key issue with enzymatically activatable prodrugs is that enzyme levels are highly heterogeneous in different patients and pathologies, as well as in different organs and (sub-)cellular compartments.

Several strategies have been explored over the years to improve the efficiency of enzyme-directed prodrug therapy (EPT) 3,4. These include prodrug-related chemical approaches, such as tailored derivatization based on spacer modification 5-7 as well as protocol-related clinical concepts. Examples of the latter are gene- (GDEPT) 8,9, virus- (VDEPT) 10-12, antibody- (ADEPT) 13-15 and polymer-directed enzyme-prodrug therapy (PDEPT) 16. While conceptually elegant, none of these approaches has resulted in translational success. This is mostly because they all require full clinical co-development of an accompanying second drug, just serving to activate the prodrug, which is not very cost-effective, and also challenging from a regulatory point of view. Furthermore, considering the different physicochemical and pharmacokinetic properties of small molecule prodrugs versus the enzyme-delivering nanoparticles, antibodies, and polymers, it is difficult to ensure a matching biodistribution, i.e., a good overlap in temporal and spatial co-localization for the prodrugs and the accompanying activating agents.

To bypass the above limitations, we here propose ultrasound-directed enzyme-prodrug therapy (UDEPT). Focused ultrasound (FUS) is nowadays applied non-invasively, in a very well-controllable manner. Its use has rapidly expanded over the years, for multiple different diseases. Particularly high-intensity focused ultrasound (HIFU) has been gaining much attention, serving as a non-invasive ablation alternative for surgical interventions and percutaneous radiofrequency ablation 17. In cancer, HIFU has been employed to induce mechanical or thermal damage to malignant tissue, thereby promoting the necrosis of tumor cells 18. From a prodrug activation point of view, we hypothesize that HIFU via mechanical destruction, a process also known as histotripsy 19,20, is an ideal modality to locally trigger the release of intact enzymes from cancer cells in a manner that is temporally and spatially tailorable and tightly controllable, without compromising enzyme activity.

**Figure 1:**
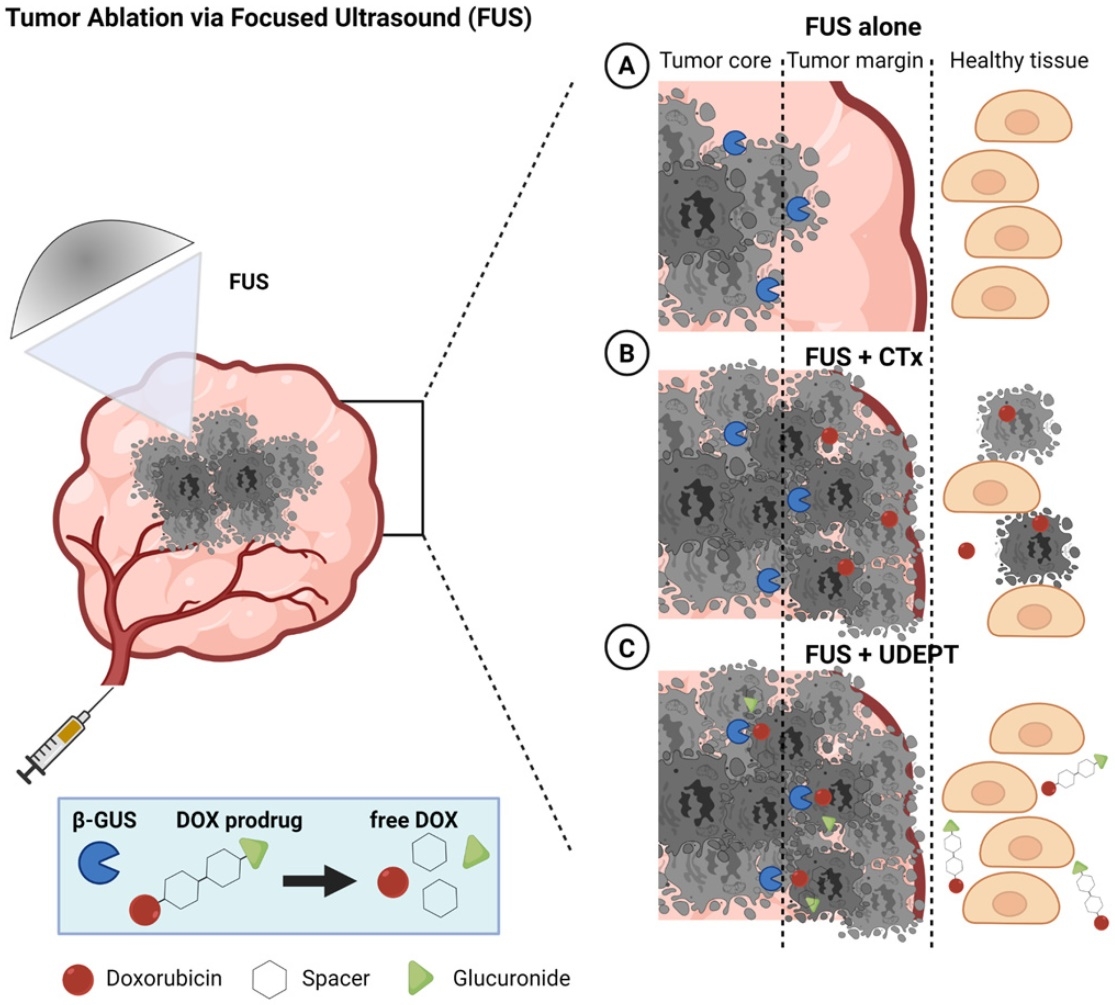
** Schematic illustration of FUS tumor ablation potentiated by UDEPT with β-GUS-sensitive self-immolative DOX prodrugs.** FUS mechanically destroys cancer cells and thereby increases the extracellular levels of β-GUS for DOX prodrug activation. **(A)** FUS alone induces cancer cell death in the tumor core and leaves the outer tumor margin and the viable rim intact, often resulting in disease relapse. **(B)** The combination of FUS with standard chemotherapy (CTx) leads to strong tumor reduction but comes with a high level of off-target toxicity in healthy tissues. **(C)** Combining FUS with UDEPT results in the destruction of the tumor core, release of β-GUS, activating prodrugs in the tumor rim, and killing cancer cells in the outer tumor margin. Off-target toxicity in healthy tissues is attenuated due to the lack of enzyme activation there. Figure generated using BioRender.

As depicted schematically in Figure 1, UDEPT is particularly useful for cleaning up the tumor margin border zones. These are located between the FUS-ablated dead tumor cores and the neighboring non-affected rims, which typically remain viable and often contain surviving cancer cells responsible for local disease relapse (Figure 1, panel 1).

In a UDEPT setup, the tumor core is mechanically destroyed by FUS, killing the tumor cells and releasing intracellular enzymes. These enzymes then diffuse locally and display their activities in the outer tumor core and in the neighboring viable margin zones, converting prodrugs into active parent drugs and killing the remaining cancer cells there. At the same time, healthy tissue surrounding the tumor and other areas in the body remain relatively unaffected (Figure 1, panel 3). This as opposed to situations in which FUS-induced tumor ablation is combined with standard chemotherapy, and in which significant healthy tissue toxicity can be expected (Figure 1, panel 2).

As a first step towards realizing UDEPT as a means to improve FUS ablation therapy, we set out to develop prodrugs that are efficiently activated by enzymes released upon ultrasound-induced mechanical cell death. We focused on β-glucuronidase (β-GUS), because β-GUS is only present within cells under physiological conditions, specifically within lysosomes. In pathological situations, e.g. in tumors with large necrotic areas, β-GUS can also be found extracellularly, although its levels are highly variable in different cancer types and tumor stages 21,22. Even though *in vivo* studies have shown successfully the use of EPT 3,4, by promoting necrotic cell death, as induced very potently via FUS-mediated mechanical cell destruction, the availability of β-GUS in the tumor extracellular space and in the tumor border margin zones is strongly increased, where it can then assist in activating prodrugs containing β-D-glucuronide as a capping group.

Based on this reasoning, we synthesized a series of self-immolative β-D-glucuronide-capped doxorubicin (DOX) prodrugs, taking pioneering previous efforts in this regard into account 23-25. We designed prodrugs to contain different spacer lengths between the anthracycline drug moiety and the β-D-glucuronide capping group, and we systematically studied the impact of prodrug design on enzyme activation kinetics. We chose to develop prodrugs based on self-immolative linkers because these have several unique features over other prodrug designs, including tunable activation kinetics 26. Our results show that spacer length affects prodrug activation kinetics, cellular uptake and cytotoxicity. They also demonstrate that β-GUS released from HIFU-destroyed cancer cells remains active, and that the β-GUS-containing cell lysate obtained upon mechanical ablation activates prodrugs in a spacer length-dependent manner. Together, our work provides promising initial proof-of-concept for the use of UDEPT-based mechanochemical combination therapies for solid tumor treatment.

## Material and Methods

### Materials

D-(+)-glucuronic acid γ-lactone, pyridine, methyl-(p-hydroxymethyl) benzoate, triethylamine (TEA), acetic acid, dichloromethane (DCM), acetonitrile (ACN), methanol (MeOH), heptane, ethyl acetate, toluene, tetrahydrofuran (THF), *N, N*-dimethylformamide (DMF) were ordered from Carl Roth. *Tert*-butyldimethylsilyl chloride (TBDMSCl), diphenyl phosphoryl azide (DPPA), lithium methoxide solution (LiOMe), 4-nitrophenyl chloroformate (Cl-COOPhNO_2_) were ordered from Sigma. Doxorubicin hydrochloride salt (DOX·HCl) was ordered from Biomol.

### Synthesis

**Scheme 1:**
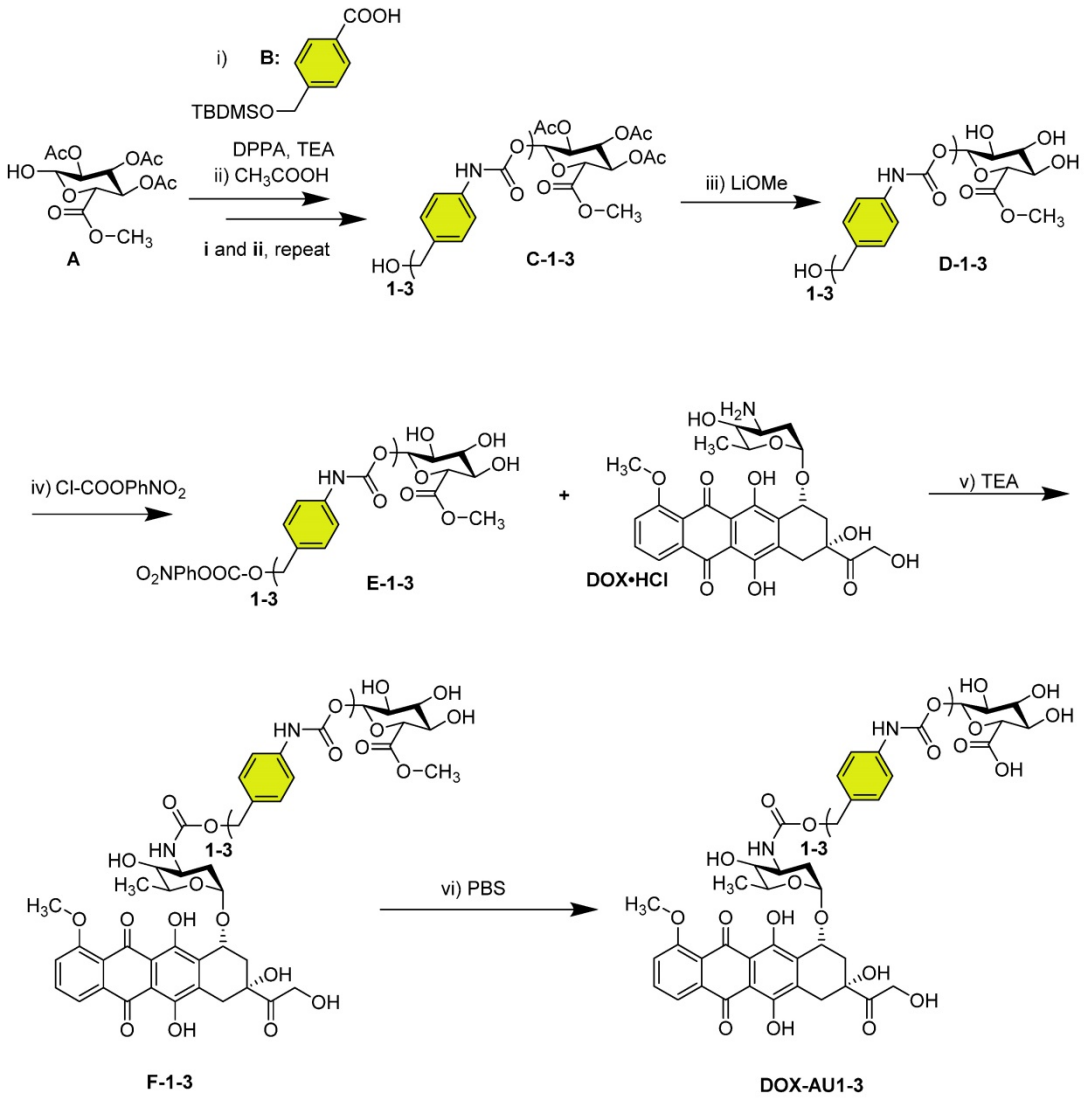
** Synthesis of glucuronide-capped self-immolative doxorubicin prodrugs.** Synthetic strategy for glucuronide-capped self-immolative DOX prodrugs containing 1-3 aromatic units (AU, colored in yellow) in the spacer structure (**DOX-AU1-3**). Precursors **D-1-3** were synthesized from the carbamate bond formation (steps *i*-*iii*) between the compound **A** and the corresponding isocyanate derivative of **B** (obtained after reaction with diphenylphosphoryl azide (DPPA) in TEA). **DOX-AU1-3** were synthesized by carbamate bond formation between the corresponding activated benzyl alcohol of **D-1-3** (**E-1-3**) and DOX·HCl in triethylamine (TEA) (steps *iv* and *v*). The final deprotection step (*vi*) was carried out at 37 °C in PBS (pH 7.4).

The synthetic route of DOX AU1-3 is depicted in Scheme 1. Detailed experimental procedure is described in the supplementary information.

### Characterization by ^1^H-NMR, ESI-MS, and HPLC

^1^H-NMR: ^1^H-NMR spectra of the synthesized compounds were recorded using a Bruker 600 FT NMR spectrometer in CD_3_OD, or CDCl_3_ or DMSO-*d*_6_ as indicated in the methods. Chemical shifts were reported as δ values (ppm) with tetramethylsilane (TMS) as the internal reference. Multiplicities were shown as s (singlet), d (doublet), t (triplet), m (multiplet); coupling constants (*J*) were displayed in Hertz (Hz), rounded to the nearest 0.1 Hz. ESI-MS: Full spectrometry was recorded with ThermoFisher LTQ-Orbitrap XL, positive ion mode, m/z (rel. intensity %). Reversed-phase analytical HPLC: HPLC analyses were measured in a C18 column, with a gradient elution method (40% ACN / 60% H_2_O + 0.1% TFA to 95% ACN / 5% H_2_O + 0.1% TFA in 11 minutes). The flow rate was 1 mL/min and the detection wavelength, 486 nm.

### Activation of DOX-AU1-3 by β-GUS

DOX-AU1-3 were dissolved in DMSO to prepare 1 mM stock solutions, which were diluted in PBS (pH 7.4 or 6.5) containing β-GUS (activity ≥1,000 units/mg, ordered from Sigma) to yield mixtures of 50 μM DOX-AU1-3 and 50 μg/mL β-GUS in the solutions. Afterwards, the mixtures were kept at 37 °C, and 200 μL of samples were taken out from the solutions at scheduled time points. The samples were analyzed by HPLC to detect the concentrations of DOX-AU1-3 and DOX. All the experiments were performed in triplicates. HPLC conditions: C18 column; gradient elution method (40% ACN / 60% H_2_O + 0.1% TFA to 95% ACN / 5% H_2_O + 0.1% TFA in 11 minutes). The flow rate was 1 mL/min, and the detection wavelength of 486 nm.

### Cell culture

Mouse mamma carcinoma (4T1, ATCC CRL-2539) and mouse embryo fibroblast (NIH/3T3, ATCC CRL-1658) were obtained from ATCC (Rockville, MD, USA) and cultured in RPMI 1640 and Dulbecco's Modified Eagle's Medium (DMEM), respectively (Sigma). The medium was supplemented with 10% Fetal Bovine Serum (Sigma F7524) and 1% penicillin-streptomycin-amphotericin B (Sigma). Cells were cultured at 37 °C in 5% CO_2_ in an air humidified incubator and regularly tested negative for mycoplasma contamination.

### Uptake and cytotoxicity of DOX, DOX-AU1, and DOX-AU2

To determine the cellular uptake, compounds were prepared at a concentration of 25 µM in the corresponding culture media. 4T1 cells were cultured in 24-well plates (200,000 cells/well) on coverslips and incubated overnight. The next day, the media was removed, and the compounds were added in media supplemented with β-GUS (50 μg/mL) or PBS. After incubation, for 4 hours at standard culture conditions, the cells were fixed with a 4% (v/v) formaldehyde solution for 10 minutes at room temperature (RT). The fixed cells were stained with a mixture of 4′,6-diamidino-2-phenylindole (DAPI), and wheat germ agglutinin (WGA) Alexa Fluor™ 488 conjugate each diluted in PBS 1:500 and incubated for 10 minutes at RT. After every step, the cells were washed once with PBS. After mounting the coverslips with Mowiol on glass microscopy slides, pictures were taken with an Axio Imager M2 microscope (Carl Zeiss Microimaging GmbH, Germany) and analyzed with ImageJ. The median signal intensity was determined, and statistical analyses were performed with GraphPad Prism 9.

For cell viability studies, working concentrations of compounds (final amount <1% DMSO) were prepared in the corresponding cell culture media. Cells were cultured in a 96-well plate (5,000 cells/well) overnight and incubated with the compounds in 200 µL culture media at concentrations ranging between 0.01 and 100 µM. The medium was supplemented with either β-GUS (50 μg/mL) or PBS. After incubation for 72 h at standard culturing conditions, cell survival was measured by 2,3-bis-(2-methoxy-4-nitro-5-sulfophenyl)-2*H*-tetrazolium-5-carboaanilide (XTT) assay according to the manufacturer protocol. Each assay was performed per triplicate. The same protocol was followed for the pulse incubation experiments, but cells were incubated with the compounds for 4 h. After that time, the supernatant was removed, cells washed, and supplemented with new media. Cells were incubated further for another 68 h before XTT assay was performed for cell survival measurement. Finally, cell viability was calculated as the percentage of viable cells compared to the untreated control cells.

### HIFU setup

High-intensity focused ultrasound (HIFU) exposure was performed by an in-house-built system consisting of a transducer, oscilloscope, combined amplifier and wave generator, and sample holder. The single element focused ultrasound transducer (Imasonic, Besançon, France) had a focal length of 8 cm, an external radius of the aperture of 14 cm, and a focal point of 1×1×3 mm^3^ (at -3dB). Sine-shaped waves were generated by an AG Series Amplifier (AG 1006, T&C Power Conversion Inc.) operated at a frequency of 1.3 MHz, duty cycle of 1%, and a pulse repetition time of 50 ms. The oscilloscope measured the input voltage of the transducer. These input voltages were used to determine acoustic pressures calibrated in the focal points as a function of input voltage using a fiber-optic probe hydrophone (FOPH 2000, RP Acoustics, Leutenbach, Germany) in a tank filled with degassed water.

### HIFU treatment of 4T1 cells

4T1 cells were added in a PCR tube (Bio-rad, California USA) (2·10^6^ cells for microscopy (Section 10); 8.5 × 10^6^ cells for prodrug activation, cytotoxicity, and calreticulin translocation study (see below Section 11, 12 and 13)). The PCR tube was positioned in the sample holder in the focus of the ultrasound beam for 10 minutes and exposed to HIFU at a peak negative pressure (p^-^) of 41 MPa. Upon HIFU exposure, a vortex was generated, leading to homogenous exposure of HIFU to all cells in the suspension. After HIFU exposure, samples containing cells were immediately placed on ice. Subsequently, the sample was analyzed by microscopy or centrifuged at 16,000 g for 15 minutes, and the supernatant was used in prodrug activation and cell studies. For the freeze-thaw procedure used as a control, 8.5 × 10^6^ cells were placed in tubes and immersed in liquid nitrogen for 2 minutes. After that time, they were removed and incubated at 37 °C for 4 minutes. The cycle was repeated three times before enzyme quantification.

### β-GUS activity by MUG assay

The β-GUS activity was investigated by 4-methyl-umbelliferyl-β-D-glucuronide (MUG) assay adapted from Jefferson et al. 27. Briefly, 20 µL sample was added to 180 µL 4-MUG (1 mg/mL in 0.1 M sodium acetate pH 4.5) and incubated for 1 h in a water bath of 37 °C. Subsequently, 950 µL of 0.2 M sodium carbonate (i.e. stopping buffer) was added to 50 µL of all samples. Finally, fluorescence intensity was measured using a spectrofluorometer (Jasco FP8300) with excitation of 380 nm and emission of 454 ± 5 nm.

### Activity of β-GUS exposed to HIFU

Bovine β-GUS (8.5 µg in 170 µL PBS corresponding to 19 units in 170 µL) exposed to HIFU with a) different peak-negative pressures in the range of 0 to 41 MPa for 10 minutes and b) a peak-negative pressure of 41 MPa for exposure durations up to 20 minutes. The β-GUS activity was measured by a MUG assay. The β-GUS activity was normalized to untreated enzymes. All experiments were performed in triplicate, and error bars represent standard deviation.

### Bright-field microscopy

After exposure of 4T1 cells to HIFU treatment, a peak-negative pressure of 41 MPa for 20 minutes, 10 µL sample, and 240 µL cell culture medium were placed in an ibidi chamber of 1µ-Slide 8 Well ibiTreat (Ibidi GmbH, Munich, Germany) and incubated for 60 minutes under standard cell culture conditions. Finally, cells were imaged by inverted bright field microscopy (Olympus CK2, ULWCD 0.30, Japan) with a Moticam 5-5.0 MP camera using a 20× objective.

### Activation of DOX-AU2 in the presence of HIFU-treated cell lysate

The experimental procedure of DOX-AU2 activation by HIFU-treated 4T1 cell supernatant was similar to that by commercial β-GUS. Firstly, 4T1 triple-negative breast cancer cells were treated by HIFU as described before to release intracellular β-GUS, which was mixed with 1 mM DOX-AU2 in DMSO. The mixture was diluted with PBS to reach DOX-AU2 at 50 μM. The activation study was performed at 37 °C, and the drug concentrations were analyzed by HPLC.

### Cytotoxicity of DOX-AU2 in the presence of HIFU-treated cell lysate

4T1 cells were cultured in a 96-well plate (5,000 cells/well) overnight and then incubated with DOX-AU2 or DOX in 200 µL culture media at concentrations between 0.01 and 100 µM. The medium was supplemented with either 20 µL of HIFU-treated cell lysate supernatant or PBS. After incubation for 72 hours at standard culturing conditions, cell survival was measured by XTT assay according to the manufacturer protocol. Finally, cell viability was calculated as the percentage of viable cells compared to the untreated control cells.

### Calreticulin translocation analysis by flow cytometry

4T1 cells were cultured in 12-well plates (100000 cells/well) and treated the next day with doxorubicin, or doxorubicin prodrug with and without HIFU treated cell lysate for 18 hours. The respective IC50 concentrations of the compounds were used. After the treatment, the cells were collected (detached by trypsin) and washed three times with cold PBS, and then stained with anti-calreticulin primary antibody (Calreticulin (D3E6) XP^®^ Rabbit mAb, #12238 from Cell Signaling Technology) and Alexa647 secondary antibody (Anti-rabbit IgG (H+L), F(ab')2 Fragment (Alexa Fluor® 647 Conjugate), #4414 from Cell Signaling Technology). After that, the cells were washed with cold PBS and stained with Hoechst 33342 (for detecting dead cells) for 5 minutes before analysis by flow cytometry (BD FACSCanto II).

### Statistical analysis

All data are presented as mean with error bars representing standard deviation. GraphPad Prism9 was used to perform the statistical analysis, and significance was determined either by unpaired t-test, one-way or two-way ANOVA with multiple comparison. A p-value of less than 0.05 was defined as statistically significant.

## Results and Discussion

We synthesized three glucuronide-based DOX prodrugs, containing 1, 2 and 3 aromatic units (AU, color-coded in yellow) within their structure (**DOX-AU1-3**, **Scheme 1**). Precursor synthesis started by repeated carbamate linkage and subsequent acidic deprotection steps between the anomerically unprotected glycosyl **A** and the corresponding isocyanate derivative of **B** to render **C-1-3** (steps *i* and *ii*, **Scheme 1**). Subsequently,** C-1-3** were deprotected in basic conditions (**D-1-3**, step *iii*,** Scheme 1**) and reacted with 4-nitrophenyl chloroformate (Cl-COOPhNO_2_) to yield **E-1-3** (step *iv*, **Scheme 1**). The glucuronide-protected DOX prodrugs (**F-1-3**) were prepared through carbamate linkage between the amino group of DOX and the activated benzyl alcohol group of the corresponding glucuronide-spacer precursors **E-1-3** (step *v*, **Scheme 1**).

The synthesis of self-immolative DOX prodrugs has been a long-standing challenge in prodrug chemistry. This is because DOX readily decomposes under the basic conditions that are typically employed in the final removal of the hydroxyl and the carboxyl protecting groups of the sugar moiety 28-30. This results in multiple side-products, in difficulties in purification, and in relatively low yields. Strategies to overcome these limitations have e.g. included the use of other protecting groups, such as allyl groups, which can be removed without the need for basic conditions 31-33. Also these approaches, however, rendered relatively low yields, and they furthermore gave rise to several byproducts 31.

The synthetic strategy presented here provides two crucial improvements in the modular preparation of a self-immolative DOX prodrug platform: (1) the deprotection of the acetyl groups of the carbohydrate before DOX coupling; and (2) the use of mild conditions, i.e. PBS buffer at pH 7.4, for the final methyl ester hydrolysis of **F-1-3** (step *vi*, **Scheme 1**). Employing these synthetic refinements, we managed to obtain the prodrugs **DOX-AU1-3** in higher yields and with less byproduct formation than for previously reported protocols 30. Details on **DOX-AU1-3** synthesis and characterization are provided as Supplementary Information.

**Figure 2:**
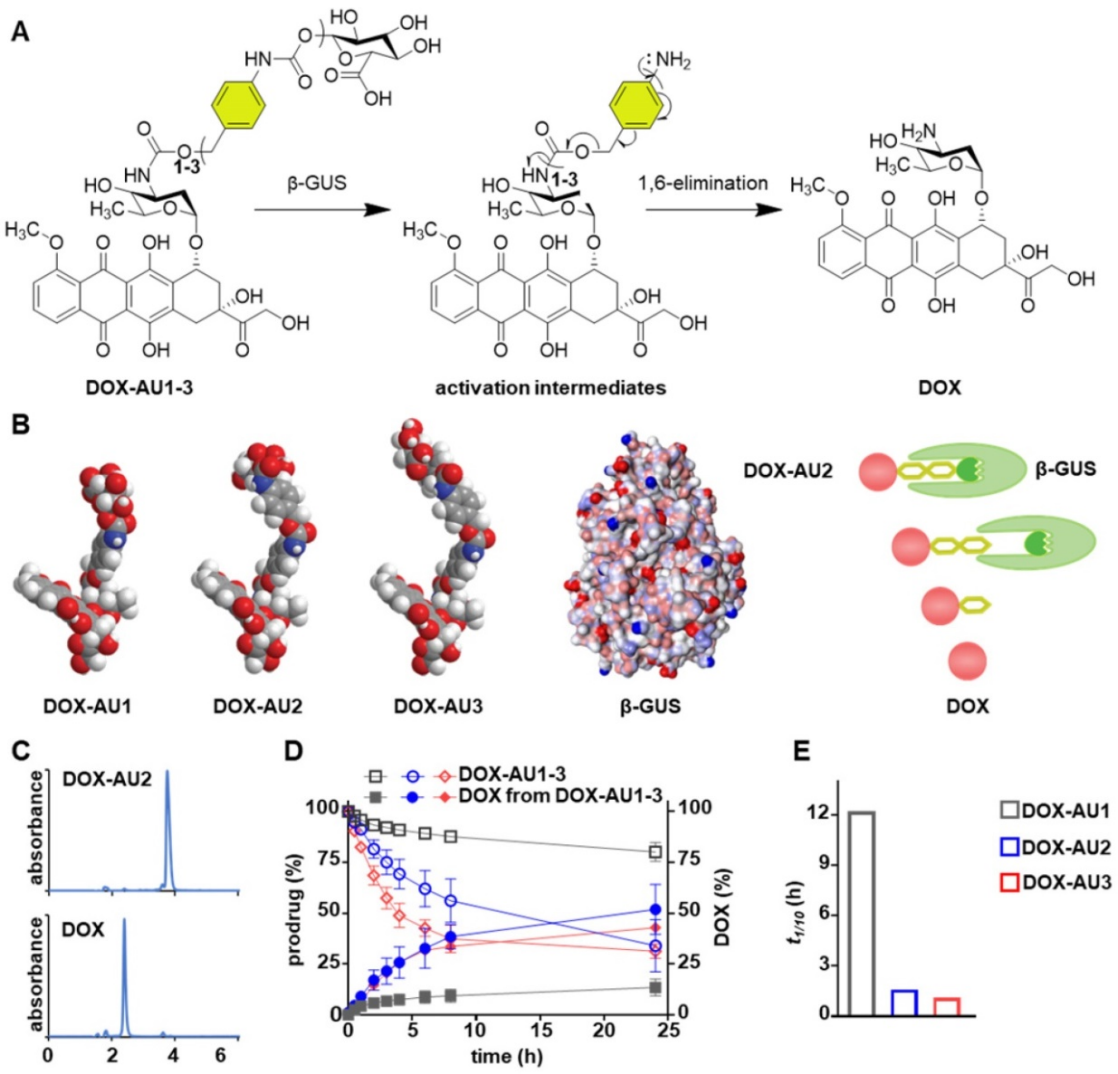
** Enzymatic activation of glucuronide-capped self-immolative doxorubicin prodrugs. (A)** β-GUS-mediated conversion of **DOX-AU1-3** to the parent drug DOX. β-GUS first digests the glucuronide moiety in the prodrugs, followed by self-immolation of the spacer via elimination of the aromatic units. **(B)** Increasing the spacer length by adding more aromatic units reduces steric hindrance. It facilitates the insertion of the glucuronide moiety into the catalytic pocket of β-GUS, resulting in faster and more efficient prodrug activation. **(C)** HPLC chromatograms of **DOX-AU2** and DOX after enzymatic activation. **(D)** Kinetics of **DOX-AU1-3** degradation and DOX generation upon exposure to β-GUS at pH 7.4 and 37 °C. Enzymatic activation was significantly faster for prodrugs with spacers containing more than one aromatic unit. **(E)** The time needed to generate 10% (*t_1/10_*) of DOX upon enzyme exposure was significantly shorter for prodrugs with spacers containing more than one aromatic unit. Due to the very slow activation of **DOX-AU1**, *t_1/10_* was analyzed instead of *t_1/2_*.

The DOX prodrugs **DOX-AU1-3** are selectively cleaved by β-GUS, triggering self-immolation of the spacer and release of native DOX (**Figure 2A**). We hypothesized that prolonging the spacer leads to improved insertion of the glucuronide moiety into the catalytic pocket of β-GUS and that this enhanced access results in increased prodrug activation kinetics (**Figure 2B**). To study this, we monitored the **DOX-AU1-3** conversion in the presence of β-GUS at 37 °C and physiological pH by high-performance liquid chromatography (HPLC; **Figure 2C**). The results in **Figure 2D** clearly show that increasing the spacer length between DOX and the β-D-glucuronide capping group significantly improved prodrugs activation and DOX generation (**DOX-AU3** > **DOX-AU2** >** DOX-AU1**). DOX generation upon enzymatic activation was approximately ten times faster for **DOX-AU2** and **DOX-AU3** than for **DOX-AU1** (**Figure 2E**). The difference in DOX generation kinetics between **DOX-AU2** and **DOX-AU3** was marginal (**Figure 2E**). We hypothesize that there is a threshold of spacer length below which the catalytic center of β-GUS cannot be efficiently accessed, and that beyond this threshold, further prolonging the spacer does not further facilitate enzymatic activation of prodrugs. This explains our finding that more than one aromatic unit is needed to enable efficient β-GUS-mediated prodrug activation, and that extending the length of the spacer from two to three aromatic groups does not add much value. Based on this notion, **DOX-AU2** was employed in the remainder of the experiments reported below for direct comparison with **DOX-AU1** and DOX.

**Figure 3:**
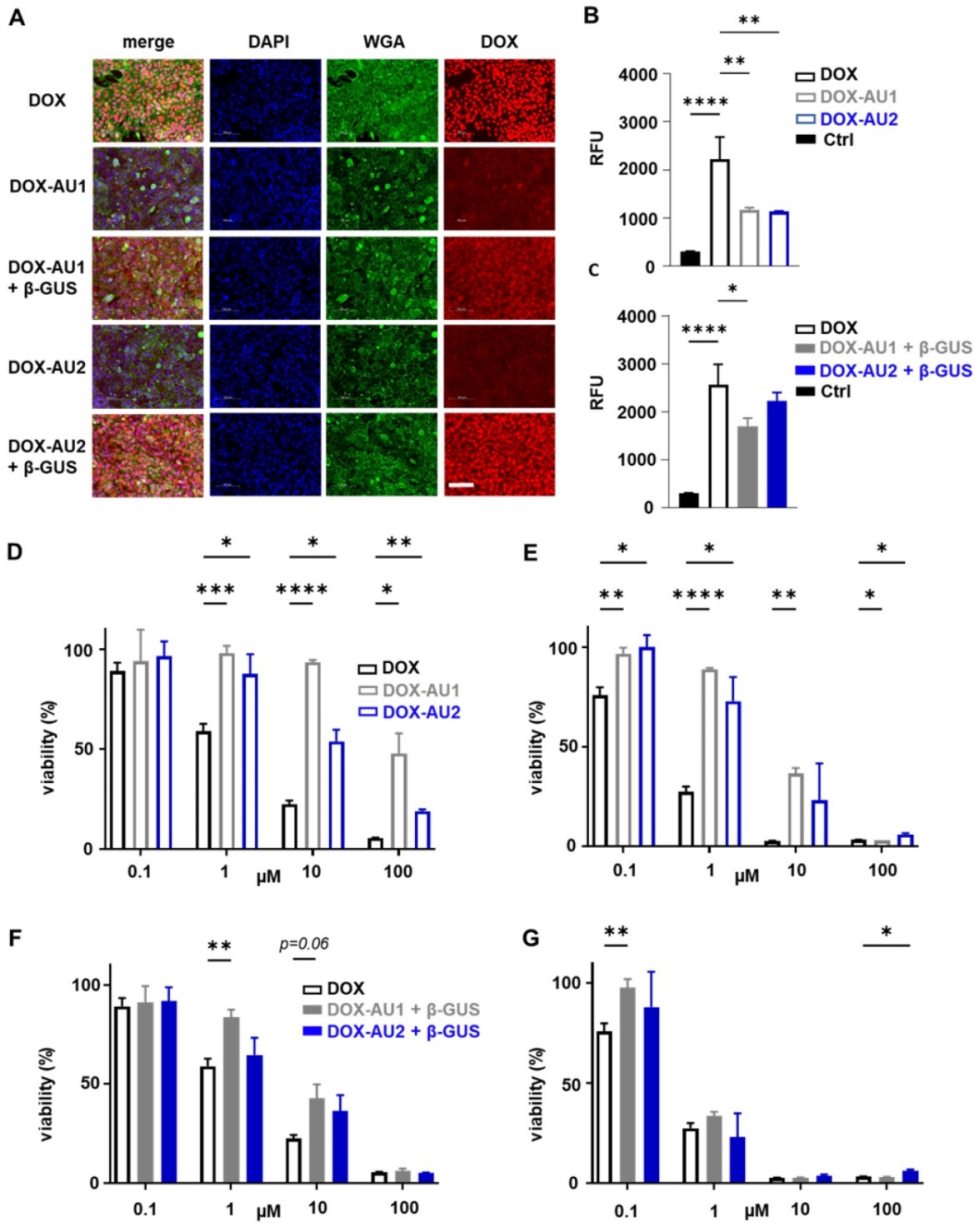
** Cellular uptake and cytotoxicity of DOX and DOX prodrugs. (A)** Fluorescence microscopy images of 4T1 breast cancer cells incubated with 25 µM of DOX, **DOX-AU1**, and **DOX-AU2** for 4 h in the presence and absence of β-GUS (scale bar 100 µM). **(B-C)** Quantification of intracellular fluorescence for DOX and DOX prodrugs in the presence and absence of β-GUS compared to untreated cells (Ctrl). **(D-G)** Viability of 4T1 breast cancer cells treated with DOX and DOX prodrugs pulse-incubated for 4 h (followed by 68 h incubation in medium; D, F) and incubated continuously for 72 h (E, G), in the presence (F, G) and absence (D, E) of β-GUS. Statistical differences were determined using a one- way (B, C) and two-way ANOVA with multiple comparison (D-G). *p ≤ 0.05, **p ≤ 0.01, ***p ≤ 0.001, ****p ≤ 0.0001.

We employed fluorescence microscopy to study the cellular internalization of the prodrugs and DOX. This was done in 4T1 breast cancer cells upon 4 h of incubation with or without β-GUS. As expected, DOX itself was rapidly internalized and present mainly in the nucleus (**Figure 3A**). In the absence of β-GUS, the intracellular fluorescence of cells treated with **DOX-AU1** and** DOX-AU2** was significantly lower than those treated with DOX (**Figure 3A-B**). This demonstrates that avoidance of rapid cellular uptake is one of the mechanisms via which the prodrug design suppresses DOX cytotoxicity. In the presence of β-GUS, the intracellular fluorescence intensity increased for **DOX-AU1** and** DOX-AU2** (**Figure 3A**). This increase was more prominent for **DOX-AU2** than for **DOX-AU1**, which is in line with the faster enzymatic activation kinetics of the former (**Figure 3C**).

To compare the *in vitro* anticancer activity of **DOX-AU1** and **DOX-AU2** to that of DOX, two sets of cytotoxicity experiments were performed, based on short pulse incubation (4 h with compounds, then 68 h with medium only) and prolonged continuous incubation (72 h with compounds). The prodrugs' cytotoxic activity was evaluated in 4T1 breast cancer cells and NIH/3T3 fibroblasts, both in the absence and presence of β-GUS. The results demonstrate that the prodrug design successfully reduces DOX-mediated cytotoxicity in both cell lines within a range of concentrations (**Figures 3D-E** and **Figure S1**). In the case of pulse incubation in the absence of enzyme, **DOX-AU2** was ~10-fold less active and **DOX-AU1** ~100-fold less active than DOX (**Figure 3D**). Upon incubation with enzyme, both prodrugs became significantly more cytotoxic, with **DOX-AU2** being more active than **DOX-AU1**, particularly at short incubation times and low concentrations (**Figure 3F-G**). Similar findings were observed in NIH/3T3 fibroblasts (**Figure S1**). These results are in line with the faster activation kinetics observed for prodrugs containing more than one aromatic unit in the spacer (**Figure 2D-E**).

**Figure 4:**
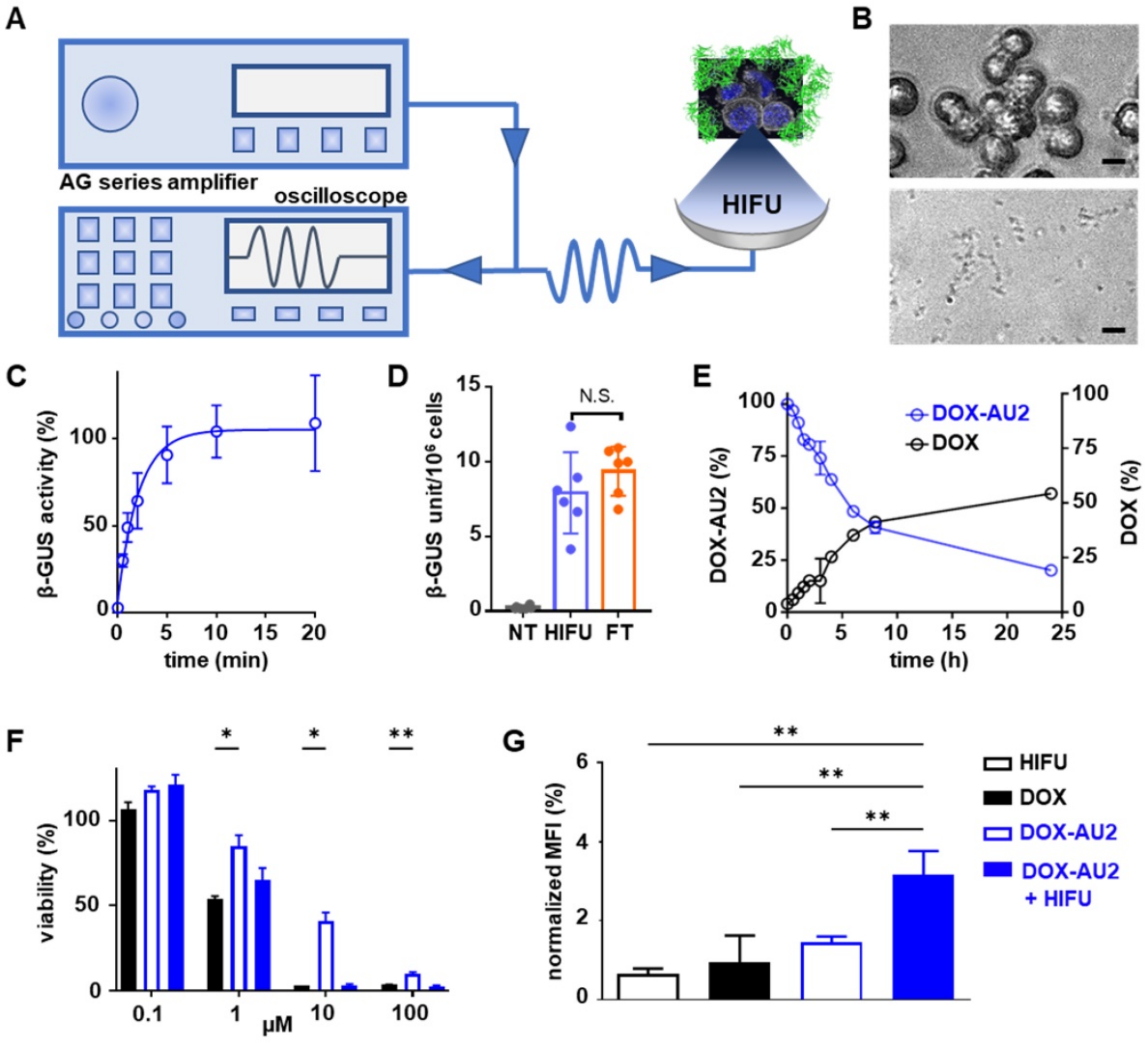
** Focused ultrasound-induced mechanical cell destruction induces β-GUS release and promotes prodrug activation. (A)** Schematic HIFU setup, composed of an amplifier/generator and an oscilloscope. The HIFU transducer spatially focuses the US energy and mechanically destroys tumor cells to release intracellular β-GUS into the environment for prodrug activation. **(B)** Bright-field microscopy images of 4T1 breast cancer before (upper) and after (lower) HIFU treatment for 10 min at 41 MPa peak-negative pressure (scale bar 20 µM). **(C)** Release kinetics of β-GUS into the extracellular environment upon HIFU treatment. **(D)** Bioactivity of β-GUS released from HIFU-treated 4T1 cells as assessed by the MUG assay. Non-treated (NT) cells display very low extracellular β-GUS activity. Three cycles of freeze-thawing (FT) served as a positive control for cell lysis and β-GUS release. **(E) DOX-AU2** conversion and DOX generation by β-GUS released from HIFU-treated 4T1 cells. **(F)** Cytotoxicity of **DOX-AU2** in 4T1 cells in the presence and absence of β-GUS released from HIFU-treated 4T1 cells, showing that prodrug incubated with supernatant from HIFU-damaged cells exhibited similar cytotoxicity as parent DOX. **(G)** Flow cytometry analysis of calreticulin translocation in HIFU, DOX, DOX AU-2 and DOX AU-2 plus HIFU-lysate -treated 4T1 cells, showing significant amounts of calreticulin translocated to the outer surface of the cell membrane for prodrug plus HIFU-lysate, indicating induction of immunogenic cell death. Statistical differences were determined using two-way ANOVA with multiple comparison (F) and unpaired *t*-test (D, H). N.S. (non-significant), *p ≤ 0.05, **p ≤ 0.01.

We next set out to demonstrate that DOX prodrug activation is promoted by HIFU-induced mechanical cell destruction, which results in the liberation of β-GUS from the lysosomal compartments of cells into the extracellular environment (**Figure 4A**). To this end, we first determined the optimal HIFU parameters to liberate cellular β-GUS. A range of different ultrasound exposure times and peak-negative pressures were applied to a cell suspension, and they were tested in terms of β-GUS release (expressed in units of enzyme activity). The optimal parameters were chosen for further experiments (**Figure S2**). Microscopy imaging confirmed that 10 min of HIFU treatment at a peak-negative pressure of 41 MPa efficiently destroyed 4T1 breast cancer cells (**Figure 4B**). Release of intact and active β-GUS after HIFU treatment was verified using the 4-methyl-umbelliferyl-β-D-glucuronide (MUG) assay, demonstrating the maximal release of the active enzyme within 10 min of HIFU treatment (**Figure 4C**). The β-GUS level in the extracellular environment was significantly higher for HIFU-treated cells than for non-treated control cells, and similar to that of cells lysed by three cycles of freeze-thawing (FT; which served as a positive control for complete cell destruction; **Figure 4D**). Importantly, HIFU-exposure did not significantly elevate the temperature of the solution, thus ensuring that the enzymatic activity of β-GUS was preserved (**Figure S3 and S4**). Furthermore, the peak-negative pressure and the time of HIFU treatment did not significantly affect β-GUS activity (**Figure S5**).

Subsequently, the supernatant of 4T1 breast cancer cells mechanically destroyed by HIFU was added to** DOX-AU2**. HPLC analysis confirmed efficient prodrug conversion (**Figure 4E**). In this context, it needs to be considered that β-GUS is stable in cell lysate, and that conversion activity over time thus only depends on the number of cells mechanically destroyed by HIFU. To assess the *in vitro* efficacy of the prodrug in the presence of cell-derived β-GUS, the HIFU-treated cell lysate was mixed with the prodrug and the mixture was added to 4T1 cells. As shown in **Figure 4F**, the prodrug combined with β-GUS-containing cell lysate from HIFU-treated cells induced a comparable level of cytotoxicity as native DOX, and it was significantly more active than the non-activated prodrug. Finally, since DOX is a potent inducer of immunogenic cell death (ICD; a mechanism that helps to induce anti-tumor immune responses), we also examined the ability of HIFU to induce enzyme release with subsequent **DOX-AU2** activation to trigger calreticulin translocation, which is a key feature of ICD 34,35. As expected, upon β-GUS-mediated prodrug activation, **DOX-AU2** induced a prominent increase in calreticulin translocation to the outer surface of the cell membrane (**Figure 4G-H**).

## Conclusion

Taken together, we here describe a modular synthetic strategy to obtain β-GUS-activatable self-immolative DOX prodrugs with different spacer lengths. Our synthetic protocol overcomes known hurdles in the design of self-immolative DOX prodrugs. Our results demonstrate that elongation of the self-immolative spacer beyond one aromatic group increases the prodrug's activation rate and *in vitro* anticancer activity. We also show that HIFU-induced cell destruction releases intact β-GUS from cancer cells, thereby enabling local UDEPT-based activation of glucuronide-based prodrugs. Such setups and strategies hold promise for the rational design of prodrugs and prodrug-based combination therapies, including mechanochemical means to improve patients' responsiveness to immunotherapy.

## Supplementary Material

Supplementary figures and experimental details.
