# Supplementary material for: Ultrasound-directed enzyme-prodrug therapy (UDEPT) using self-immolative doxorubicin derivatives
Source: Theranostics. 2022 Jun 6;12(10):4791–801. doi: 10.7150/thno.69168 (PMC9254251; doi:10.7150/thno.69168)
Supplement: Supplementary file 1 — Supplementary figures and experimental details. [file thnov12p4791s1.pdf]

# - Supplementary Information -

Ultrasound-directed enzyme-prodrug therapy (UDEPT) using self-immolative doxorubicin derivatives

Karolin Roemhild[‡], Helena C. Besse[‡], Bi Wang[‡], Quim Peña, Qingxue Sun, Daiki Omata, Burcin Ozbakir, Clemens Bos, Hans W. Scheeren, Gert Storm, Josbert M. Metselaar, Haijun Yu, Ruth Knüchel-Clarke, Fabian Kiessling, Chrit T.W. Moonen, Roel Deckers*, Yang Shi*, Twan Lammers*

CONTENTS:

1. EXPERIMENTAL DETAILS 2

2. SUPPLEMENTARY FIGURES 5

3. SUPPLEMENTARY REFERENCES 10

1. EXPERIMENTAL DETAILS

Synthetic procedures:

Compound A. Compound A was synthesized according to a previous publication [1,2]. Yield: 17.3% (mixture of alpha and beta sugars). ^{1}H NMR (600 MHz, DMSO-d_6) δ 7.61 – 7.37 (m, 1H, -OH), 5.53 – 5.12 (m, 2H, Glu 1,3-H), 5.12 – 4.86 (m, 1H, Glu 2-H), 4.86 – 4.58 (m, 1H, Glu 4-H), 4.41 (d, *J* = 10.2 Hz, 1H, Glu 5-H), 3.64 (s, 3H, $COOCH_3$), 2.03 – 1.88 (m, 9H, -OAc).

Compound B. Compound B synthesis was performed using a previous established method [3]. Yield: 57.5%. ^{1}H NMR (600 MHz, DMSO-d_6): δ 12.85 (s, 1H, -COOH), 7.90 (d, *J* = 8.2 Hz, 2H, Ar), 7.38 (d, *J* = 8.0 Hz, 2H, Ar), 4.77 (s, 2H, Ar-CH_2), 0.91 (s, 9H, Si-tBu), 0.08 (s, 6H, Si-Me_2).

Compounds C-1, C-2 and C-3. Firstly, compound B (HOOC-Ph-CH_2-O-TBDMS) (6 g, 1 eq) was dissolved in anhydrous toluene (120 mL) under argon atmosphere, and triethylamine (TEA, 1.2 eq) and diphenylphosphoryl azide (DPPA, 1.2 eq) were added. The reaction mixture was heated to 85 °C for 3 h with stirring. When this reaction mixture was cooled down to room temperature, compound A (0.8 eq) in anhydrous toluene (60 mL) was added and the mixture was stirred overnight. After evaporation of the solvent, the residue was purified by column chromatography (heptane/ethyl acetate = 5/2) to get TBDMS protected single spacer moiety. Afterwards, removal of the tert-butyldimethylsilyl protecting group (TBDMS) was performed in THF/H_2O/acetic acid = 1/1/1 for 4 hours. The reaction mixture was concentrated, followed by purification via column chromatography (heptane/ethyl acetate = 2/3) to get C-1. Yield: 65.4%. For the syntheses of C-2 and C-3, the above procedures were repeated for 1 and 2 more times, respectively (pure TBDMS-protected C-2 and C-3 were obtained by silica column chromatography using heptane/ethyl acetate = 2/3 and 2/1, respectively). Yield of C-2, C-3: 38.3%; 23.7%. ^{1}H NMR (600 MHz, $CDCl_3$); compound C-1: δ 7.41 – 7.29 (m, 4H, Ar), 6.95 (s, 1H, NH), 5.78 (d, *J* = 8.0 Hz, 1H, Glu 1-H), 5.34 (t, *J* = 9.4 Hz, 1H, Glu 2-H), 5.24 (t, *J* = 9.7 Hz, 1H, Glu 3-H), 5.17 (dd, *J* = 9.3 Hz, 1H, Glu 4-H), 4.65 (s, 2H, Ar-CH_2), 4.20 (d, *J* = 9.8 Hz, 1H, Glu 5-H), 3.74 (s, 3H, $COOCH_3$), 2.08 – 2.01 (m, 9H, OAc). Compound C-2: δ 7.43 – 7.28 (m, 8H, Ar), 7.02 (s, 1H, NH), 6.70 (s, 1H, NH), 5.78 (d, *J* = 8.0 Hz, 1H, Glu 1-H), 5.35 (t, *J* = 9.4 Hz, 1H, Glu 2-H), 5.25 (t, *J* = 9.6 Hz, 1H, Glu 3-H), 5.19 (dd, *J* = 9.3 Hz, 1H, Glu 4-H), 5.14 (s, 2H, Ar-CH_2), 4.64 (s, 2H, Ar-CH_2), 4.21 (d, *J* = 9.8 Hz, 1H, Glu 5-H), 3.74 (s, 3H, $COOCH_3$), 2.08 – 2.02 (m, 9H, OAc). Compound C-3: δ 7.47 – 7.28 (m, 12H, Ar), 7.05 (s, 1H, NH), 6.77 (s, 1H, NH), 6.71 (s, 1H, NH), 5.78 (d, *J* = 8.1 Hz, 1H, Glu 1-H), 5.35 (t, *J* = 9.4 Hz, 1H, Glu 2-H), 5.25 (t, *J* = 9.6 Hz, 1H, Glu 3-H), 5.19 (dd, *J* = 9.3 Hz, 1H, Glu 4-H), 5.14 (s, 2H, Ar-CH_2), 5.13 (s, 2H, Ar-CH_2), 4.64 (s, 2H, Ar-CH_2), 4.20 (d, *J* = 9.9 Hz, 1H, Glu 5-H), 3.73 (s, 3H, $COOCH_3$), 2.08 – 2.02 (m, 9H, OAc).

Compounds D-1, D-2 and D-3. A solution of C-1, or C-2, or C-3 (4.1 mmol) in anhydrous MeOH (50 mL) was cooled down to 0 °C. To this solution, 1 M LiOMe (4.6 mL) in MeOH (45 mL) was added dropwise. After 2 hours, the reaction mixture was neutralized by adding silica and the crude purified by silica column chromatography (2% MeOH/ethyl acetate followed by 4% MeOH/ethyl acetate) after filtration and concentration. Yield of D-1, D-2, D-3: 53.6%; 58.0%; 25.1%. ^{1}H NMR (600 MHz, $MeOD_4$); compound D-1: δ 7.49 – 7.24 (m, 4H, Ar), 5.49 (d, *J* = 8.0 Hz, 1H, Glu 1-H), 4.55 (s, 2H, Ar-CH_2), 3.97 (d, *J* = 9.7 Hz, 1H, Glu 5-H), 3.77 (s, 3H, $COOCH_3$), 3.56 (t, *J* = 9.3 Hz, 1H, Glu 2-H), 3.48 (t, *J* = 9.1 Hz, 1H,

Glu 3-H), 3.42 (dd, *J* = 9.1 Hz, 1H, Glu 4-H). Compound D-2: δ 7.52 – 7.21 (m, 8H, Ar), 5.50 (d, *J* = 8.0 Hz, 1H, Glu 1-H), 5.12 (s, 2H, Ar-CH_2), 4.53 (s, 2H, Ar-CH_2), 3.98 (d, *J* = 9.7 Hz, 1H, Glu 5-H), 3.77 (s, 3H, $COOCH_3$), 3.56 (t, *J* = 9.3 Hz, 1H, Glu 2-H), 3.49 (t, *J* = 9.0 Hz, 1H, Glu 3-H), 3.43 (dd, *J* = 9.1 Hz, 1H, Glu 4-H). Compound D-3: δ 7.63 – 7.14 (m, 12H, Ar), 5.50 (d, *J* = 8.0 Hz, 1H, Glu 1-H), 5.12 (s, 2H, Ar-CH_2), 5.11 (s, 2H, Ar-CH_2), 4.53 (s, 2H, Ar-CH_2), 3.97 (d, *J* = 9.7 Hz, 1H, Glu 5-H), 3.77 (s, 3H, $COOCH_3$), 3.56 (t, *J* = 9.3 Hz, 1H, Glu 2-H), 3.48 (t, *J* = 9.0 Hz, 1H, Glu 3-H), 3.42 (dd, *J* = 9.1 Hz, 1H, Glu 4-H).

Compounds E-1, E-2 and E-3. D-1, or D-2, or D-3 (1 mmol) was dissolved in a mixture of ACN (32 mL) and THF (4.3 mL). After cooling the mixture down to 0 ^{o}C, pyridine (1.3 eq) and 4-nitrophenyl chloroformate (Cl-COOPh·NO_2, 1.2 eq) were added. The reaction was stirred for 2 hours. Afterwards, the crude was purified by silica column chromatography (8% MeOH in DCM) to afford compound E-1, or E-2, or E-3. Yield of E-1, E-2, E-3: 71.1%; 78.4%; 52.0%. ^{1}H NMR (600 MHz, $MeOD_4$); compound E-1: δ 8.39 – 8.23 (m, 2H, Ar-NO_2), 7.58 – 7.36 (m, 6H, Ar), 5.52 (d, *J* = 8.1 Hz, 1H, Glu 1-H), 5.25 (s, 2H, Ar-CH_2), 4.02 (d, *J* = 9.7 Hz, 1H, Glu 5-H), 3.76 (s, 3H, $COOCH_3$), 3.58 (t, *J* = 9.3 Hz, 1H, Glu 2-H), 3.50 (t, *J* = 9.0 Hz, 1H, Glu 3-H), 3.44 (dd, *J* = 9.1 Hz, 1H, Glu 4-H). Compound E-2: δ 8.41 – 8.19 (m, 2H, Ar-NO_2), 7.58 – 7.28 (m, 10H, Ar), 5.50 (d, *J* = 7.8 Hz, 1H, Glu 1-H), 5.24 (s, 2H, Ar-CH_2), 5.13 (s, 2H, Ar-CH_2), 3.98 (d, *J* = 9.6 Hz, 1H, Glu 5-H), 3.77 (s, 3H, $COOCH_3$), 3.56 (t, *J* = 9.1 Hz, 1H, Glu 2-H), 3.49 (t, *J* = 8.8 Hz, 1H, Glu 3-H), 3.43 (dd, *J* = 8.4 Hz, 1H, Glu 4-H). Compound E-3: δ 8.42 – 8.23 (m, 2H, Ar-NO_2), 7.60 – 7.28 (m, 14H, Ar), 5.51 (d, *J* = 8.0 Hz, 1H, Glu 1-H), 5.24 (s, 2H, Ar-CH_2), 5.12 (s, 2H, Ar-CH_2), 5.11 (s, 2H, Ar-CH_2), 4.01 (d, *J* = 9.7 Hz, 1H, Glu 5-H), 3.77 (s, 3H, $COOCH_3$), 3.57 (t, *J* = 9.3 Hz, 1H, Glu 2-H), 3.49 (t, *J* = 9.0 Hz, 1H, Glu 3-H), 3.43 (dd, *J* = 9.1 Hz, 1H, Glu 4-H).

Compounds F-1, F-2 and F-3. Compound E-1, or E-2, or E-3 (1.2 mmol) was dissolved in anhydrous DMF (34 mL) under N_2. DOX·HCl (1 mmol) and TEA (1 mmol) were added to this solution. The reaction was kept on stirring overnight, and the DMF was removed. The crude product was purified by silica column chromatography (12% MeOH in ethyl acetate) to yield F-1, F-2 and F-3. Yield of F-1, F-2, F-3: 71.9%; 96.1%; 91.2%. ^{1}H NMR (600 MHz, DMSO-d_6); compound F-1: δ 14.04 (s, 1H, 6-OH), 13.29 (s, 1H, 11-OH), 9.96 (s, 1H, Ar-NH), 7.95 – 7.90 (m, 2H, Ar), 7.72 – 7.61 (m, 1H, Ar), 7.52 – 7.15 (m, 4H, Ar), 6.83 (d, *J* = 8.0 Hz, 1H, 3'-NH), 5.48 – 5.39 (m, 4H, 2*Glu-OH, and Glu 1-H, and 9-OH), 5.32 (d, *J* = 4.9 Hz, 1H, Glu-OH), 5.23 – 5.19 (m, 1H, 1'-H), 4.98 – 4.92 (m, 1H, 7-H), 4.87 (s, 2H, Ar-CH_2), 4.83 (t, *J* = 5.9 Hz, 1H, 14-OH), 4.68 (d, *J* = 5.9 Hz, 1H, 4'-OH), 4.57 (d, *J* = 6.4 Hz, 2H, 14-CH_2), 4.15 (d, *J* = 6.7 Hz, 1H, 5'-H), 3.99 (s, 3H, 4-OMe), 3.90 (d, *J* = 9.0 Hz, 1H, Glu-5H), 3.79 – 3.68 (m, 1H, 3'-H), 3.66 (s, 3H, $COOCH_3$), 3.49 – 3.22 (m, 4H, Glu 2,3,4-H and 4'-H), 3.04 – 2.93 (m, 2H, 10_{eq} and 10_{ax}-H), 2.23 – 2.09 (m, 2H, 8_{ax} and 8_{eq}-H), 1.88 – 1.78 (m, 1H, $2'_{ax}$-H), 1.52 – 1.41 (m, 1H, 2'eq -H), 1.12 (d, *J* = 6.4 Hz, 3H, 5'-CH_3). Compound F-2: δ 14.04 (s, 1H, 6-OH), 13.29 (s, 1H, 11-OH), 10.03 (s, 1H, Ar-NH), 9.72 (s, 1H, Ar-NH), 7.94 – 7.90 (m, 2H, Ar), 7.69 – 7.63 (m, 1H, Ar), 7.53 – 7.18 (m, 8H, Ar), 6.81 (d, *J* = 8.0 Hz, 1H, 3'-NH), 5.48 – 5.39 (m, 4H, 2*Glu-OH, and Glu 1-H, and 9-OH), 5.32 (d, *J* = 4.9 Hz, 1H, Glu-OH), 5.24 - 5.19 (m, 1H, 1'-H), 5.04 (s, 2H, Ar-CH_2), 4.99 – 4.9 (m, 1H, 7-H), 4.87 (s, 2H, Ar-CH_2), 4.83 (t, *J* = 5.9 Hz, 1H, 14-OH), 4.68 (d, *J* = 5.9 Hz, 1H, 4'-OH), 4.57 (d, *J* = 6.4 Hz, 2H, 14-CH_2), 4.15 (d, *J* = 6.7 Hz, 1H, 5'-H), 3.99 (s, 3H, 4-OMe), 3.90 (d, *J* = 9.0 Hz, 1H, Glu-5H), 3.79 – 3.68 (m, 1H, 3'-H), 3.66 (s, 3H, $COOCH_3$), 3.49 – 3.22 (m, 4H, Glu 2,3,4-H and 4'-H), 3.04 – 2.93 (m, 2H, 10_{eq} and 10_{ax}-H), 2.23 – 2.09 (m, 2H, 8_{ax} and 8_{eq}-H), 1.88 – 1.78 (m, 1H, $2'_{ax}$-H), 1.53 – 1.39 (m, 1H, 2'eq -H), 1.12 (d, *J* = 6.4 Hz, 3H, 5'-CH_3). Compound F-3: δ 14.04 (s, 1H, 6-OH), 13.29 (s, 1H, 11-OH), 10.03 (s, 1H, Ar-NH), 9.83 (s, 1H, Ar-NH), 9.72 (s, 1H, Ar-NH), 7.93 – 7.90 (m, 2H, Ar), 7.68 – 7.63 (m, 1H, Ar), 7.54 – 7.17 (m, 12H, Ar), 6.81 (d, *J* = 8.0 Hz, 1H, 3'-NH), 5.48 – 5.39 (m, 4H, 2*Glu-OH, and Glu 1-H, and 9-OH), 5.32 (d, *J* = 4.9 Hz, 1H, Glu-OH), 5.24 – 5.17 (m, 1H, 1'-H), 5.06 (s, 2H, Ar-CH_2), 5.02 (s, 2H, Ar-CH_2), 4.95 – 4.90 (m,

1H, 7-H), 4.87 (s, 2H, Ar-CH_2), 4.77 (t, J = 5.9 Hz, 1H, 14-OH), 4.68 (d, J = 5.9 Hz, 1H, 4'-OH), 4.57 (d, J = 6.4 Hz, 2H, 14-CH_2), 4.15 (d, J = 6.7 Hz, 1H, 5'-H), 3.99 (s, 3H, 4-OMe), 3.90 (d, J = 9.0 Hz, 1H, Glu-5H), 3.79 – 3.68 (m, 1H, 3'-H), 3.66 (s, 3H, $COOCH_3$), 3.49 – 3.22 (m, 4H, Glu 2,3,4-H and 4'-H), 3.04 – 2.93 (m, 2H, 10_{eq} and 10_{ax}-H), 2.23 – 2.09 (m, 2H, 8_{ax} and 8_{eq}-H), 1.88 – 1.78 (m, 1H, $2'_{ax}$-H), 1.51 – 1.44 (m, 1H, 2'eq -H), 1.12 (d, J = 6.4 Hz, 3H, 5'-CH_3).

g) *Compounds DOX-AU1, DOX-AU2 and DOX-AU3.* F-1, or F-2, or F-3, respectively, (1 mmol) were dissolved in DMSO and PBS (v/v=1/1) and stirred at 37 °C for 20 hours. Solvent was removed by lyophilization, and the crude was purified by reversed-phase HPLC to obtain DOX-AU1-3. Yield of DOX-AU1-3: 71.6%; 78.3%; 72.5%.

DOX-AU1: ^{1}H NMR (600 MHz, DMSO-d_6) δ 14.04 (s, 1H, 6-OH), 13.29 (s, 1H, 11-OH), 9.96 (s, 1H, Ar-NH), 7.95 – 7.90 (m, 2H, Ar), 7.72 – 7.61 (m, 1H, Ar), 7.52 – 7.15 (m, 4H, Ar), 6.83 (d, J = 8.0 Hz, 1H, 3'-NH), 5.49 – 5.32 (m, 4H, 2*Glu-OH, and Glu 1-H, and 9-OH), 5.27 (d, J = 5.2 Hz, 1H, Glu-OH), 5.23 – 5.19 (m, 1H, 1'-H), 4.98 – 4.92 (m, 1H, 7-H), 4.88 (s, 2H, Ar-CH_2), 4.83 (t, J = 5.9 Hz, 1H, 14-OH), 4.68 (d, J = 5.7 Hz, 1H, 4'-OH), 4.57 (d, J = 6.4 Hz, 2H, 14-CH_2), 4.15 (d, J = 6.9 Hz, 1H, 5'-H), 3.99 (s, 3H, 4-OMe), 3.72 (d, J = 9.1 Hz, 1H, Glu-5H), 3.64 – 3.57 (m, 1H, 3'-H), 3.49 – 3.22 (m, 4H, Glu 2,3,4-H and 4'-H), 3.09 – 2.88 (m, 2H, 10_{eq} and 10_{ax}-H), 2.23 – 2.09 (m, 2H, 8_{ax} and 8_{eq}-H), 1.87 – 1.77 (m, 1H, $2'_{ax}$-H), 1.52 – 1.41 (m, 1H, 2'eq -H), 1.12 (d, J = 6.5 Hz, 3H, 5'-CH_3). MS (ESI$^+$, MeOH): $[M+Na]_{cal}$ = 935.8; $[M+Na]_{find}$ = 935.2. Reversed-phase analytical HPLC (40% ACN / 60% H_2O + 0.1% TFA to 95% ACN / 5% H_2O + 0.1% TFA in 11 minutes): R_t = 3.02 min (Purity: 86%).

DOX-AU2: ^{1}H NMR (600 MHz, DMSO-d_6) δ 14.04 (s, 1H, 6-OH), 13.29 (s, 1H, 11-OH), 10.02 (s, 1H, Ar-NH), 9.72 (s, 1H, Ar-NH), 7.95 – 7.89 (m, 2H, Ar), 7.69 – 7.63 (m, 1H, Ar), 7.53 – 7.18 (m, 8H, Ar), 6.81 (d, J = 8.1 Hz, 1H, 3'-NH), 5.54 – 5.35 (m, 4H, 2*Glu-OH, and Glu 1-H, and 9-OH), 5.28 (d, J = 5.0 Hz, 1H, Glu-OH), 5.24 – 5.19 (m, 1H, 1'-H), 5.04 (s, 2H, Ar-CH_2), 4.99 – 4.9 (m, 1H, 7-H), 4.87 (s, 2H, Ar-CH_2), 4.84 (t, J = 5.9 Hz, 1H, 14-OH), 4.68 (d, J = 5.6 Hz, 1H, 4'-OH), 4.57 (d, J = 6.4 Hz, 2H, 14-CH_2), 4.15 (d, J = 6.9 Hz, 1H, 5'-H), 3.99 (s, 3H, 4-OMe), 3.75 (d, J = 9.3 Hz, 1H, Glu 5-H), 3.73 – 3.66 (m, 1H, 3'-H), 3.49 – 3.22 (m, 4H, Glu 2,3,4-H and 4'-H), 3.09 – 2.88 (m, 2H, 10_{eq} and 10_{ax}-H), 2.24 – 2.09 (m, 2H, 8_{ax} and 8_{eq}-H), 1.88 – 1.77 (m, 1H, $2'_{ax}$-H), 1.53 – 1.39 (m, 1H, 2'eq -H), 1.12 (d, J = 6.5 Hz, 3H, 5'-CH_3). MS (ESI$^+$, MeOH): $[M+Na]_{cal}$ = 1084.9; $[M+Na]_{find}$ = 1084.3. Reversed-phase analytical HPLC (40% ACN / 60% H_2O + 0.1% TFA to 95% ACN / 5% H_2O + 0.1% TFA in 11 minutes): R_t = 3.79 min (Purity: 88%).

DOX-AU3: ^{1}H NMR (600 MHz, DMSO-d_6) δ 14.02 (s, 1H, 6-OH), 13.28 (s, 1H, 11-OH), 10.04 (s, 1H, Ar-NH), 9.83 (s, 1H, Ar-NH), 9.75 (s, 1H, Ar-NH), 7.93 – 7.90 (m, 2H, Ar), 7.68 – 7.63 (m, 1H, Ar), 7.54 – 7.17 (m, 12H, Ar), 6.86 (d, J = 8.1 Hz, 1H, 3'-NH), 5.66 – 5.40 (m, 4H, 2*Glu-OH, and Glu 1-H and 9-OH), 5.36 (d, J = 5.2 Hz, 1H, Glu-OH), 5.24 – 5.17 (m, 1H, 1'-H), 5.06 (s, 2H, Ar-CH_2), 5.02 (s, 2H, Ar-CH_2), 4.95 – 4.90 (m, 1H, 7-H), 4.86 (s, 2H, Ar-CH_2), 4.77 (t, J = 5.9 Hz, 1H, 14-OH), 4.75 (d, J = 5.6 Hz, 1H, 4'-OH), 4.58 (d, J = 6.4 Hz, 2H, 14-CH_2), 4.14 (d, J = 6.8 Hz, 1H, 5'-H), 3.98 (s, 3H, 4-OMe), 3.70 (d, J = 9.1 Hz, 1H, Glu 5-H), 3.64 – 3.57 (m, 1H, 3'-H), 3.49 – 3.22 (m, 4H, Glu 2,3,4-H and 4'-H), 3.04 – 2.94 (m, 2H, 10_{eq} and 10_{ax}-H), 2.22 – 2.09 (m, 2H, 8_{ax} and 8_{eq}-H), 1.87 – 1.78 (m, 1H, $2'_{ax}$-H), 1.51 – 1.44 (m, 1H, 2'eq -H), 1.11 (d, J = 6.5 Hz, 3H, 5'-CH_3). MS (ESI$^+$, MeOH): $[M+Na]_{cal}$ = 1234.1; $[M+Na]_{find}$ = 1233.3. Reversed-phase analytical HPLC (40% ACN / 60% H_2O + 0.1% TFA to 95% ACN / 5% H_2O + 0.1% TFA in 11 minutes): R_t = 4.55 min (Purity: 77.5%).

2. SUPPLEMENTARY FIGURES

Figure S1:

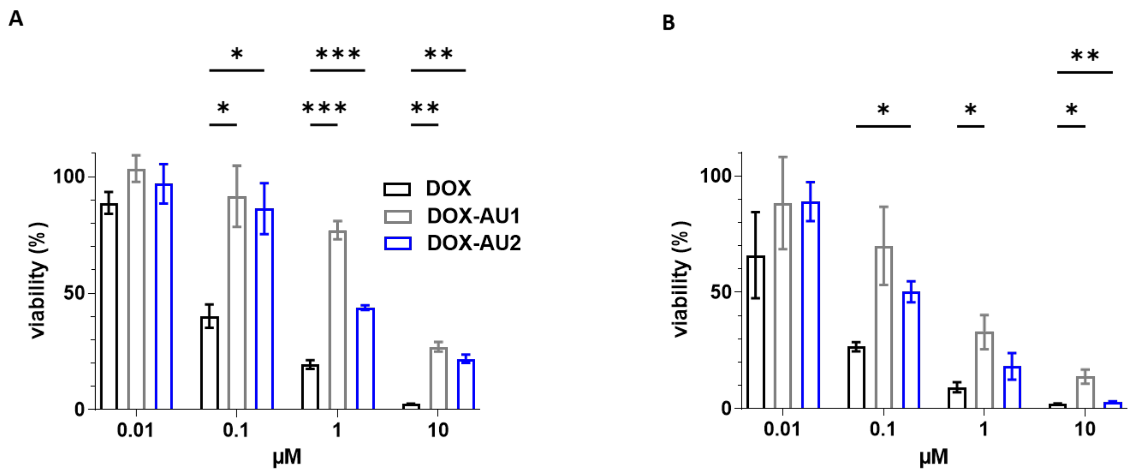

Figure S1. (A)+(B) Viability of NIH/3T3 treated with DOX and DOX prodrugs (DOX-AU1-2) incubated for (A) 4 h followed by 68 h with media alone or (B) 72 h. Statistical differences were determined using a two-way ANOVA test, with p values as: *$p \leq 0.05$, **$p \leq 0.01$, ***$p \leq 0.001$, ****$p \leq 0.0001$.

Figure S2:

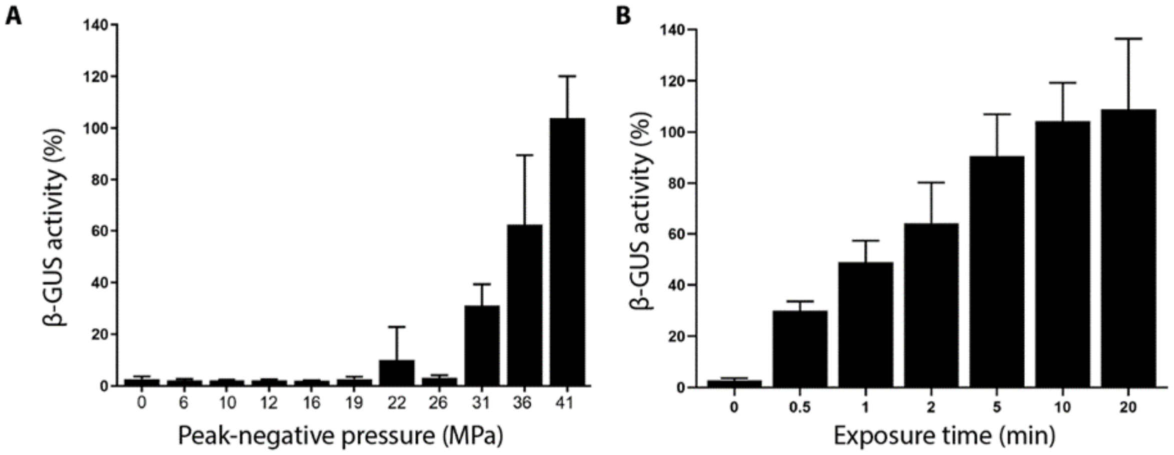

Figure S2. Effect of HIFU treatment parameters ((A) pressure, at a fixed exposure time of 10 minutes; and (B) exposure time at a fixed peak-negative pressure at 41 MPa) on β-GUS liberation expressed as enzyme activity.

Figure S3:

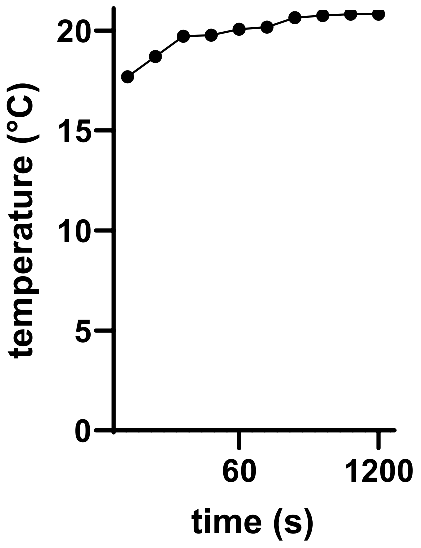

Figure S3. Influence of HIFU (41 MPa peak-negative pressure) exposure time on temperature (PBS).

Figure S4:

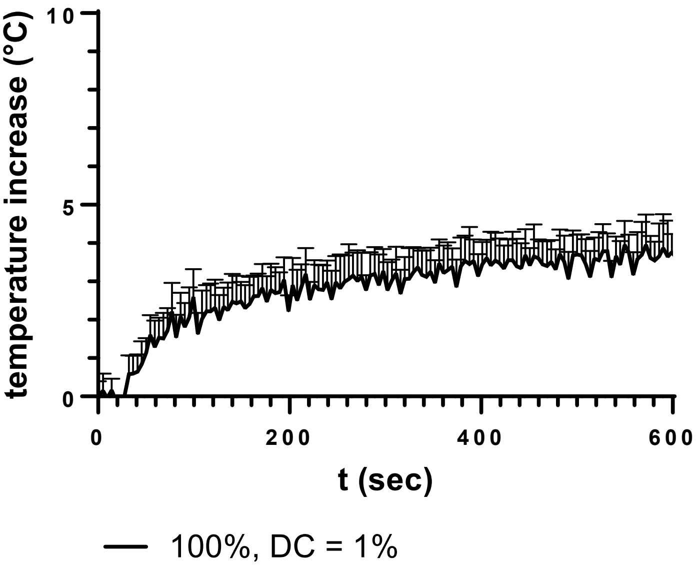

Figure S4. Influence of HIFU (41 MPa peak-negative pressure) exposure on agar gel phantom. Temperature increase was determined by MR thermometry.

Figure S5:

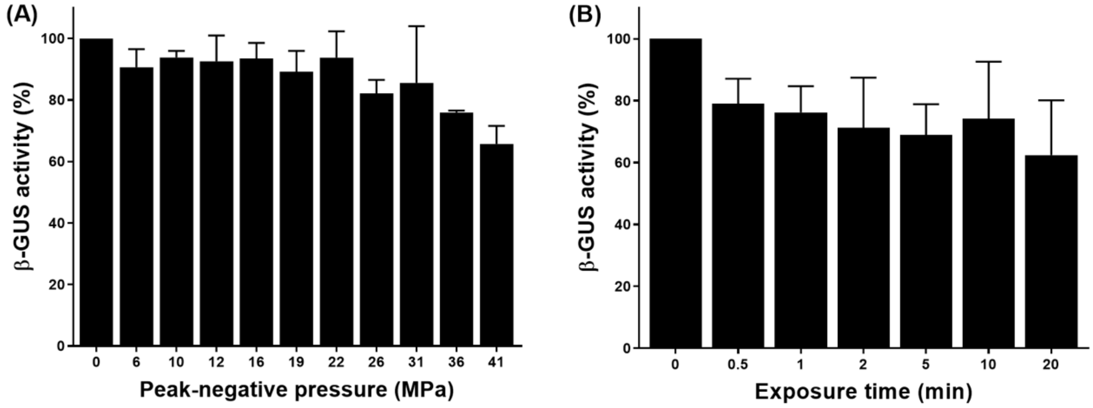

Figure S5. Influence of HIFU treatment parameters (i.e., pressure (A) and exposure time (B)) on the enzymatic activity of β-GUS.

3. SUPPLEMENTARY REFERENCES:

[1] Bosco M, Rat S, Dupré N, et al. Lewis-Acidic Polyoxometalates as Reusable Catalysts for the Synthesis of Glucuronic Acid Esters under Microwave Irradiation. ChemSusChem. 2010;3:1249–1252.

[2] Bollenback GN, Long JW, Benjamin DG, et al. The Synthesis of Aryl-D-glucopyranosiduronic Acids1. J Am Chem Soc. 1955;77:3310–3315.

[3] Groot FMH de, Albrecht C, Koekkoek R, et al. “Cascade-Release Dendrimers” Liberate All End Groups upon a Single Triggering Event in the Dendritic Core. Angewandte Chemie International Edition. 2003;42:4490–4494.
